# Health-Assistive Smart Homes for Aging in Place: Leading the Way for Integration of the Asian Immigrant Minority Voice

**DOI:** 10.31372/20180304.1087

**Published:** 2018

**Authors:** Connie Kim Yen Nguyen-Truong, Roschelle L. Fritz

**Affiliations:** a College of Nursing in Vancouver, Washington State University, Vancouver, WA, USA

**Keywords:** Asians, immigrants, minorities, health-assistive smart homes, community-engaged research, technology, artificial intelligence

## Abstract

Caring for America’s aging population is a complex humanitarian issue. The number of older adults is expected to increase to 98.5 million by 2060 with a 295% growth in foreign-born older adults, including Asian immigrants. Most older adults will have one or more chronic conditions and 95% of healthcare costs will be attributed to caring for these conditions. Among Asian Americans, common chronic conditions include respiratory disease, cancer, cardiovascular disease, and pain. The National Institutes of Health, Institute on Aging, and National Science Foundation call for innovative technologies to be developed by multidisciplinary teams to address these concerns. Asian community leaders at Asian Health & Service Center and community members in Oregon identified the use of health-assistive technologies as a priority for potentially reducing stress and improving quality of life for both older adults and their caregivers. The purpose of this article is to introduce nurses and healthcare workers, advocating for the interests of Asian/Pacific Island community members, to the innovative health-assistive smart home. The health-assistive smart home uses artificial intelligence to identify and predict health events. Inclusion of minority persons’ data in the development of artificial intelligence has been generally overlooked. This may result in continued health inequities and is incompatible with the goals of global health. Integration of minority voices while exploring the efficacious use of the health-assistive smart home is of significant value to minority populations. Asian immigrant older adults engaging in smart home research and development will enhance the cultural and technical safety of future devices. Asian families may be particularly interested in smart homes for extending independence because they place an emphasis on collective culture and family-based care. Community engagement of stakeholders and steadfast leadership are needed so that future technologies used in healthcare delivery are both technically and culturally sound. A community-engaged research approach promotes community empowerment that is responsive to community identified priorities and is a good fit for studying adoption of smart home monitoring for health-assistance.

Health-assistive smart homes using artificial intelligence (AI) are a hi-technology solution designed to aid in the care of the older adult population and to facilitate aging in place. To date, the voice of minority communities has been mostly absent in the development of the smart home’s AI algorithms, potentially leading to continued health inequities for current and future generations. This is incompatible with the goals of global health. Research purposefully including Asian voices and data is needed and is of significant value to minority populations as a whole. In this article, we introduce a new technology—health-assistive smart home—and encourage minority populations to participate in health technology research so that their voice can be included in the development of AI. We are at a critical juncture.

Everyday more than 10,000 Americans celebrate their 65^th^ birthday. American older adults currently outnumber the entire population of Canada and their numbers continue to grow (Statistics Canada, 2017; United States [U.S.] Census Bureau, 2017 [based on 2015 census data]). More than 80% of older adults have one chronic condition, 68% manage two or more chronic conditions, 36% manage four or more chronic conditions, and 95% of healthcare costs for older adults are attributed to caring for these conditions (National Council on Aging, 2017). Among Asian Americans, common chronic conditions include respiratory disease, cancer, cardiovascular disease, and pain (Carlisle, 2014). The National Institutes of Health (2014, 2017), National Institute on Aging (2017), and National Science Foundation (2017) all call for multidisciplinary teams to develop innovative technologies to address these concerns. Asian community leaders at Asian Health & Service Center (AHSC, n.d.) and community members in Oregon identified the use of health-assistive technologies as a priority for potentially reducing stress and improving quality of life for both older adults and their caregivers. These leaders are interested in health-assistive technologies to enhance care and extend independence for their older adults. However, they identified the need to explore the cultural context of technology adoption and the provision of culturally safe care.

The purpose of this article is to introduce nurses and healthcare workers, advocating for the interests of Asian/Pacific Island community members, to the innovative health-assistive smart home (hereafter referred to as smart home). The smart home uses AI to identify and predict health changes so that proactive interventions can be taken by family and/or nurses. The smart home monitors persons in the privacy of their home. Therefore, knowledge of this technology may be of importance to Asian/Pacific Island communities. Community engagement on this topic is important while the smart home is still in the research and development phase. Community engagement will help to assure the design of a culturally acceptable product where the Asian immigrant minority voice has been infused into AI features. Further, nurses may be the workforce deploying smart homes to minority communities. As nurses, working on the frontline of smart home development and design, we want to illuminate smart home research and inspire minorities from all communities (Asian, Pacific Islander, and more) to engage in research with their community partners and with local universities, and to *actively and equally be at the health-technology design table*.

In our collaboration with Asian community leaders and community members, we use a bicultural lens. This involves combining a western (independent) perspective in addition to an eastern (interdependent) perspective from the home country on a continuum that needs to be considered in regards to technology adoption. Smart home features and functionality have been primarily designed by, and tested on, non-Hispanic White older adults. There is now an immediate need to purposefully include the eastern inter-dependent perspective of Asian/Pacific Island older adults for training the AI so the smart home is capable of being culturally sensitive and appropriate. The need is immediate because the eastern perspective must not be an afterthought, but an integral and purposeful part of AI features.

## The Smart Home

The smart home is a hi-technology solution that uses sensor hardware and AI software to identify and predict changes in health states in order that proactive interventions can be taken on behalf of the older adult. The smart home will: (a) potentially extend independence by identifying changes in health and proactively intervening (i.e., identification of subtle changes in condition and alerting when treatment may be needed; Ni, Hernando, & de la Cruz, 2015); (b) lower cost of care (i.e., by reducing hospitalizations and re-hospitalizations; Skubic, Guevara, & Rantz, 2015); and (c) increase quality of life for older adults (Rantz et al., 2015), including Asian immigrants.

The smart home has unobtrusive sensors that are placed on the ceiling, walls, and doors of an existing home. These sensors detect older adults’ movements in the home. Sensors include infrared motion, contact, light, temperature, and humidity. When activated, these sensors transmit their status (e.g., ON/OFF) to a computer machine and a middleware adds a sensor identifier (e.g., BedroomABed or KitchenSink or BathroomAToilet) as well as a date and time stamp. For example, a single line of sensor data for January 1, 2018 just after midnight appears as: “2018-01-01 00:00:00.822376 BathroomASink ON,” indicating the resident is in the bathroom in the night. Currently, the smart home can detect over 40 normal older adult activities of daily living with greater than 98% accuracy and we are beginning to detect abnormal motion patterns that are clinically relevant (Fritz, Corbett, Vandermause, & Cook, 2016). Clinically relevant data is identified by adding a clinician-in-the-loop for development and design. A nurse-clinician provides clinical ground truth (i.e., real-world context that informs selection of data sets and improves accuracy in training the algorithms) for data used in training the machine’s *artificial* intelligence (Fritz & Cook, 2017). The intelligent machine then learns to recognize motion patterns that are unique to the individual.

## Health Monitoring and Privacy

The smart home monitors older adults in the privacy of their home using sensor data from daily motion patterns. It can detect changes in health (Sprint, Cook, Fritz, & Schmitter-Edgecombe, 2016) and alert family and/or caregivers. However, the home is the primary place of privacy. It is the place where one is able to live out cultural values without fear of judgment.

Findings from previous studies indicate privacy is a major concern for older adults (Cook, Schmitter-Edgecombe, & Dawadi, 2015; Demiris, Hensel, Skubic, & Rantz, 2008; Fritz et al., 2016). They are also concerned that their data may not be secure and that they will become more vulnerable to exploitation, identity theft, or safety breaches (Demiris, 2009; Fritz et al., 2016). Additionally, older adults have indicated that the technology should fit the need, be easy to use, and work as intended (Demiris et al., 2008; Demiris et al., 2004), and that it should not interfere with how they choose to live out life in the privacy of their home (Fritz et al., 2016).

Nurses working with smart home technology recognize that smart home monitoring and subsequent treatments based on smart home information may disrupt culturally specific care assumptions leading to stress, conflict, and decreased quality of life (Fritz et al., 2016). For example, Fritz et al. (2016) found in a prior study (with primarily non-Hispanic White older adults) that quality of life decreases to pre-adoption levels if the smart home malfunctioned. Furthermore, several older adults indicated a need to talk to their children and that family opinions were important to decision-making with regard to adoption of smart home technology. These findings suggest the existence of a feeling of powerlessness for autonomous decision-making. There may be additional unique and cultural considerations needed for Asian older adults with regard to adoption decisions. According to the American Association of Retired Persons Executive Summary Report (2014), 42% of Asian/Pacific Island Americans are providing care for older adults versus 22% of the general population. Park and Lee (2017) found that sibling caregivers of older Korean adults had a sense of responsibility and obligation to take care of the brother or sister that was not explicit in studies with western participants. In another study with older and younger Chinese American adults, both groups perceived advanced care planning was important; however, both groups believed it was taboo to initiate discussion as it may bring bad luck to individuals and may hasten death (Lee, Byon, Hinderer, & Alexander, 2017). Thus, discussion of smart homes for older adults’ health care may disrupt culturally appropriate communication between Asian older adults and their children. Asian older adults may prefer that their children monitor them for health and safety instead of a technology. Relying on a smart home may introduce distrust in the parent–child relationship and create misunderstandings with regard to the chidrens’ parental caregiving obligations. It is important that smart home features include ways to enhance parent–child relationships and connectivity.

Smart homes will be market ready in the near future yet so far older adults participating in this research have been primarily non-Hispanic White (i.e., of Northern European decent). The inclusion of minority data in smart home development is of significant value to minority populations and has potential to positively impact current and future generations. Nurse researchers are collaborating with engineers and psychologists to tenaciously include more minority persons in the data sample and in training the AI agent. Asian community leaders at AHSC, and Asian-based community health and social services center, and community members as partners in the Portland, Oregon metropolitan area are joining in this effort. These partners are interested in smart home possibilities because it could provide support for living out the cultural values of family-based home care and collectivism (Nguyen & Clark, 2014) and the desire to age in place (Fritz et al., 2016; Peek et al., 2016). Community engagement as stakeholders and steadfast leadership facilitating technically and culturally safe research is essential to assure Asian immigrant minority voices are heard and integrated into development of the smart home. Through a community-engaged approach, academic investigators Dr. Connie K. Y. Nguyen-Truong and Dr. Roschelle L. Fritz in collaboration with partners, Asian community leaders, and community members in Oregon including AHSC’s long-standing academic partner, Dr. Junghee Lee, are building cultural social capital (i.e., pooling multicultural and multilingual expertise and sharing technology and cultural resources) to facilitate this timely critical change in science.

We approach this critical juncture as collaborators, bringing four decades of combined expertise in nursing and nearly two decades of combined smart home research, community-based participatory research, and prolonged engagement with minority cultures. We are working alongside Asian community leaders and community members in Oregon, who are at the *health-technology design table*, to explore how smart homes can assist Asian immigrant older adults with aging in the place of their choosing. We embrace the tenets of community-engaged research, which promotes community empowerment and is responsive to their identified priorities (Ahmed & Palermo, 2010; Israel, Eng, Schulz, & Parker, 2012). During our decade of prolonged engagement with Asian community leaders and community members, of which two years were with AHSC on a prior community engaged study, we learned that “relationships are important” and that the honor of gaining entrée is based on trust due to a genuine motivation to have “meaningful impact on the Asian immigrant community” for sustainability. Having a shared cultural and research language includes honoring a history of deep rooted traumatic struggles and community grassroots efforts in promoting the health of the diverse Asian immigrant community. We build on prior research exploring the influence of culture on the adoption of smart home monitoring, and we seek to elicit Asian immigrant older adults’ perceptions and thoughts about being monitored by a smart home. We are facilitating empowerment by introducing immigrant older adults to the smart home through conversation and shared decision-making at AHSC, which is regarded as a trusted place for health information and support services (Nguyen-Truong et al., 2017). We believe that knowledge is power. In our conversations, we not only share our knowledge about smart homes and they share with us their cultural lens, but we co-construct knowledge because of engagement. We recognize that culture influences adoption decisions and we seek to learn from Asian immigrant older adults about willingesss, readiness, barriers, how, and whether, smart homes could be perceived as a safe technology choice for health-assistance.

The digital divide is a limitation that needs to be considered and exists in two ways with smart homes: financial status and age. Smart home research will inform policy advocacy regarding the complex humanitarian care of the growing aging population that includes minorities. It may also inform ideas regarding privacy and provision-of-care that uses AI to extend the reach of the nurse and facilitate safer family-based care. These health technologies are designed by young digital natives to be used by older adults who are not digital natives. Thus, there is a critical need for the researchers’ role to fuse the two horizons of gerontology and technology. This is an emerging science—*gerontechnology*. We are leading the way to design a culturally acceptable smart home that can be deployed in a culturally safe manner.

## Implications for Nurses

In the future, nurses may be the workforce deploying smart homes to support extending independence and health-assistance for older adults. They will need to understand smart home technology. In addition, they need to understand the influence culture has on perceptions of continuous in-home unobtrusive monitoring and the adoption of health-related technologies. The impact of this technology on privacy, power differentials, monitoring of daily activities and routines, family-engagement, and community expectations must be acknowledged. Likewise, minority community leaders and their partners must be involved in the research process so their voice is *heard and infused* into technological advancements, especially AI.

## Acknowledgments

The authors are grateful to the community leaders and members for their engagement: Holden Leung, MSW, Executive Director, and Christine Lau, MA, CACDI, Chief Operating Officer, at Asian Health & Service Center; Dr. Junghee Lee, PhD, Program Director and Associate Professor, at the School of Social Work at Portland State University; Tuong Vy Le, MS, BS, Dr. Jacqueline Leung, JD, MS, CHW, Thai Hien Nguyen, BSN, RN, and Anthony My Truong, BS, RPh, community member partners from the Asian/Pacific Island Communities. The authors are also grateful to Dr. Catherine Van Son, PhD, RN, ANEF, Associate Professor, at Washington State University College of Nursing in Vancouver, for editing assistance, and the anonymous peer reviewers for their assistance.

## Declaration of Conflicting Interests

The authors declared no potential conflicts of interest with respect to the research, authorship, or publication of this article.

## Funding

The authors disclosed receipt of the following financial support for the research, authorship, or publication of this article: Dr. Connie Kim Yen Nguyen-Truong, PhD, RN, (Alumnus PCCN), and Dr. Roschelle L. Fritz, PhD, RN, were awarded the Beta Psi Chapter of Sigma Theta Tau International – Naomi Ballard Nursing Research Award.
